# Identification of The Aberrantly Expressed LncRNAs in Hepatocellular Carcinoma: A Bioinformatics Analysis Based on RNA-sequencing

**DOI:** 10.1038/s41598-018-23647-1

**Published:** 2018-03-29

**Authors:** Hao-Tian Liao, Ji-Wei Huang, Tian Lan, Jin-Ju Wang, Bo Zhu, Ke-Fei Yuan, Yong Zeng

**Affiliations:** Department of Liver Surgery, Liver Transplantation Division, West China Hospital, Sichuan University, Chengdu, China

## Abstract

Hepatocellular carcinoma (HCC) is one of the most prevalent subtypes of liver cancer worldwide. LncRNAs have been demonstrated to be associated with the progression of HCC, but a systematic identification and characterization of their clinical roles and molecular mechanisms in HCC has not been conducted. In this study, the aberrantly expressed lncRNAs in HCC tissues were analyzed based on TCGA RNA-seq data. 1162 lncRNAs were found to be aberrantly expressed in HCC tissues, including 232 down-regulated lncRNAs and 930 up-regulated lncRNAs. The top 5 lncRNAs with the highest diagnostic accuracy were further analyzed to evaluate their clinical value and potential mechanism in HCC. Kaplan-Meier curves showed that higher expressions of DDX11-AS1 and AC092171.4 were in correlation with poorer survival in HCC patients. Significant difference was also observed when comparing the expression levels of DDX11-AS1 and SFTA1P in different clinical parameters (*p* < 0.05). GO analysis showed that genes regulated by the 5 lncRNAs were enriched in certain pathways, such as PI3K pathway. Moreover, GSEA analysis on the expression of DDX11-AS1 showed that DDX11-AS1 affected the gene expressions involved in HCC proliferation, differentiation and cell cycle, indicating an essential role of DDX11-AS1 in hepatocarcinogenesis.

## Introduction

Hepatocellular carcinoma (HCC) is one of the most common and malignant tumors in adults, which has been ranked as the second leading cause of cancer death all around the world^1^. While great achievement was made in the methods of therapy, such as surgical resection, liver transplantation, radiation therapy and chemotherapy, the 5-year survival rate of HCC subjects still remains poor, with more than 750,000 HCC subjects die each year^2^. Previous studies have identified many aberrantly expressed protein-coding genes in HCC, which led to great therapeutic improvement by providing novel drug targets for recurrent or unresectable HCC^3,4^. However, due to the lack of specific driver molecules or mutations, there are limitations in developing novel drugs for treating HCC. Thus, identification of potential therapeutic targets that participate in the development and progression of HCC is in urgent need.

Long noncoding RNAs (lncRNAs) have been recognized as transcripts longer than 200 nucleotides (nt) that are 5′ capped and 3′ polyadenylated, with limited protein-coding potential^5^. It has been reported that lncRNAs play essential roles in regulating the biological process and gene expression by diverse mechanism in a broad variety of cancers^6–8^. To date, emerging evidence has showed that lncRNAs are involved in the proliferation, invasion, metastasis and angiogenesis of HCC^9,10^. To our knowledge, no study has conducted a systematic identification and characterization of candidate lncRNAs involved in HCC, especially their clinical roles and molecular mechanisms in the development and progression of HCC.

In this study, we performed bioinformatical analyses based on high throughput RNA sequencing of HCC from The Cancer Genome Atlas database (TCGA, http://cancergenome.nih.gov/) to evaluate the clinicopathological value of lncRNAs and their underlying mechanism in HCC.

## Methods

### TCGA dataset of HCC

High throughput RNA sequencing data of HCC were downloaded from The Cancer Genome Atlas database (TCGA). Generally, 370 HCC samples and 50 adjacent non-cancerous samples with RNA-seq data from Illumina HiSeq platform were included in current study. Approval by a local ethics committee was not required since TCGA is a community resource project and all data in TCGA can be used for publication without restrictions or limitations. Meanwhile, this study was performed in accord with the TCGA publication guidelines (http://cancergenome.nih.gov/).

### Analysis of the aberrantly expressed lncRNAs in HCC

The RNA-Seq data of HCC form TCGA contains over 14000 lncRNAs defined by NCBI (https://www.ncbi.nlm.nih.gov/) or Ensembl (http://asia.ensembl.org/). The R package DESeq was used to normalize the expression level of each lncRNA^11^. LncRNAs of which expression was less than 1 in over 10% of the patients were excluded from this study. Then the differentially expressed lncRNAs between HCC samples and paired adjacent normal tissues were determined by using the statistical methods of DESeq, in which genes with Benjamini-Hochberg adjusted *p* value < 0.05 and absolute log2 fold-change >2 were considered differentially expressed.

### Clinical role of the top 5 aberrantly expressed lncRNAs in HCC

The area under the receiver-operating-characteristic (ROC) curve was used to assess the diagnostic accuracy of all the aberrantly expressed lncRNAs, with 1 indicating perfect discriminatory value and 0.5 or less indicating no discriminatory value. The different expressions of lncRNAs between HCCs and adjacent normal tissues, and between patients with different clinical manifestations were analyzed by using Student’s t test. Kaplan-Meier survival curves were constructed using the expressions of these 5 lncRNAs from TCGA transcriptional profiles (Statistical ranking for the normalized expression of each lncRNA by the top quartile and bottom quartile were defined as high-expression and low-expression, respectively.) as threshold and compared by log-rank analysis. All analyses were performed by using SPSS version 22.0 and *p* < 0.05 was considered statistically significant.

### Potential functions of the 5 aberrantly expressed lncRNAs in HCC

Weighted Gene Co-Expression Network Analysis (WGCNA) was used to identify modules of co-expressed genes regulated by these 5 lncRNAs^12^. The co-expression network established by WGCNA was then visualized by Cytoscape 3.5.1. In addition, Gene Ontology (GO) analysis for the 5 lncRNAs and their co-expressed genes was performed based on the Database for Annotation, Visualization and Integrated Discovery (DAVID, https://david.ncifcrf.gov/). GO terms with Benjamini-Hochberg adjusted *p* value < 0.05 were considered statistically significant.

### Gene set enrichment analysis

GSEA was performed using the GSEA-R, a bioconductor implementation of GSEA from Broad Institute^13^. The GSEA analysis was performed to compare transcriptome profiles of both high (top 25th quartile normalized expression) and low (bottom 25th quartile normalized expression) expressions of DDX11-AS1. Before GSEA analysis, normalization of expression data and generation of transcriptome profiles were performed using the R package Deseq^11^. Gene sets were obtained from published gene signatures in the Molecular Signatures Database (MSigDB). Analysis was run with 1,000 permutations and a classic statistic. Normalized enrichment score (NES) and Benjamini-Hochberg adjusted *p* values were measured to find enrichments with statistical significance (<0.05).

### Validation of the aberrantly expressed lncRNAs based on clinical samples

For further validation of our findings, the expression levels of the 5 lncRNAs were also determined in clinical samples (n = 20) from West China Hospital of Sichuan University by using qRT-PCR. All patients and their relatives provided the informed consents, and this study was approved by the Ethical Committee of Sichuan University. The samples used in this study are unselected, non-consecutive, historically confirmed HCCs without pretreatment, that were resected at West China Hospital. Following partial hepatectomy, approximately 5 g segments of tumor and the adjacent normal tissue (at least 1 cm from the surgical margin) were isolated and immediately stored in liquid nitrogen for further analysis. Total RNA was extracted using Trizol reagent (Invitrogen). First-strand cDNA was generated using the PrimeScript RT reagent Kit (Takara) according to the manufacturer’s protocol. Real-time qPCR was performed in the CFX Connect Real-Time PCR Detection System (Bio-Rad) by using SYBR Green (Takara) and the gene-specific primers. Relative transcript levels of target genes were normalized to GAPDH mRNA levels. RNA Quality Assessment was conducted by acrylamide gel electrophoresis, in which a 28S: 18S rRNA ratio of 2:1 is representative of good-quality RNA. The primers used were listed as following: GAPDH, F: 5′-TGAAGGTCGGAGTCAACGGATTT-3′, R: 5′-GCCATGGAATTTGCCATGGGTGG-3′; AC092171.4, F: 5′-ATTACCCCGCCCTGGATTTG-3′, R: 5′-TTGTTTTCCCCACCCC-3′; DDX11-AS1, F: 5′-TTAGGAGGACAACGAATCACCTC-3′, R: 5′-GTCATCTCCCAGAACCAGACTTT-3′; HAGLR, F: 5′-GATCCCCACCTTCCCCAAAG-3′, R: 5′-TCTCCGACTGAGGTTTGCAC-3′; HAGLROS, F: 5′-AGGCTGAGCGCTAACTGAAG-3′, R: 5′-TTGCCCTGTCTTCAGAGGTG-3′; SFTA1P, F: 5′-TGGGAAATGCGGATATAGAAGGT-3′, R: 5′-GATGAGCTTCCACGGATTTTCAC-3′.

### Data Availability

All data generated or analysed during this study are included in this published article.

## Results

### Identification of candidate lncRNAs based on TCGA dataset of in HCC

**Figure 1:**
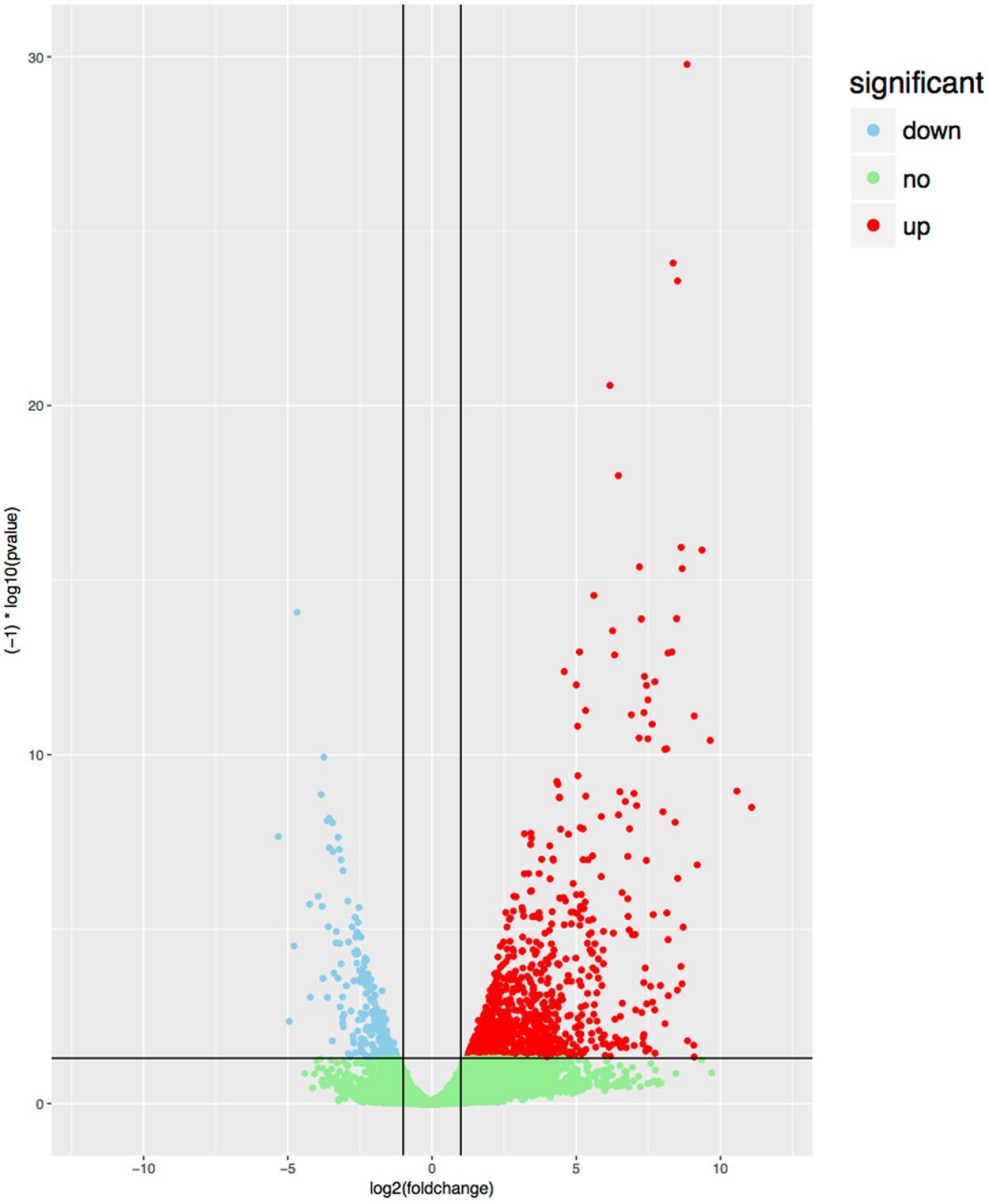
Volcano plot of the aberrantly expressed lncRNAs between HCC and adjacent normal tissues. The x-axis indicates the log2 fold change in gene expression, which was defined as the ratio of normalized value of gene expression detected in HCC and adjacent normal tissues. The y-axis indicates the adjusted *p* values plotted in −log10. Red dots represent up-regulated lncRNAs in HCC, while blue dots represent those down-regulated.

**Figure 2:**
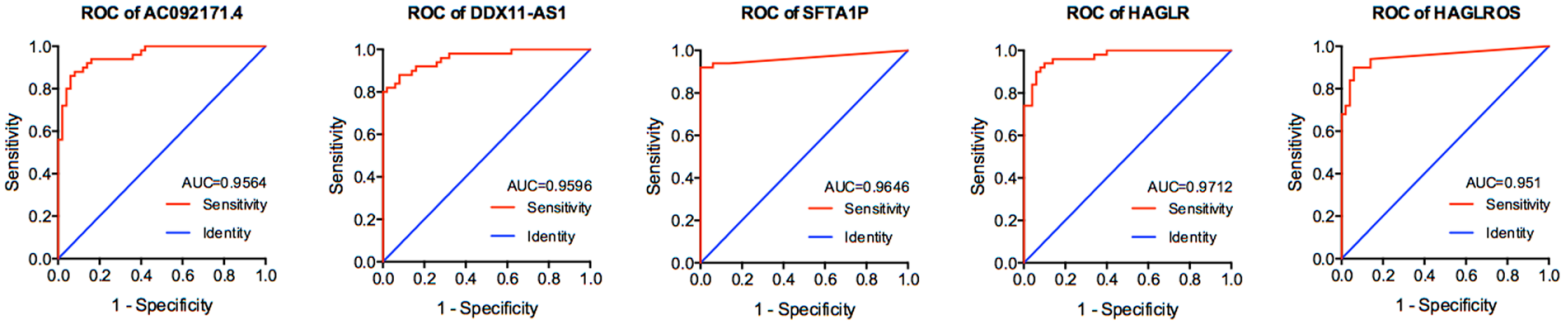
ROC curves of the top 5 lncRNAs sorted by AUC. Red line represents the sensitive curve, while green represents the identify line. The x-axis indicates false positive rate, which is presented as “1-Specificity”. The y-axis indicates true positive rate, which is presented as “Sensitivity”.

To systematically identify the aberrantly expressed lncRNAs in HCC, the expression levels of lncRNAs in 50 HCC samples and paired adjacent normal tissues from TCGA dataset were analyzed by using the R package DEseq. Finally, 1162 lncRNAs were found to be aberrantly expressed in HCC tissues, including 232 down-regulated lncRNAs and 930 up-regulated lncRNAs (Fig. 1). 5 lncRNAs with area under ROC curve (AUC) over 0.95 were selected for further analysis due to their potential roles in hepatocarcinogenesis and significant diagnostic value for patients with HCC, including HOXD antisense growth-associated long non-coding RNA (HAGLR), HAGLR opposite strand (HAGLROS), Surfactant associated 1 (SFTA1P), DDX11 antisense RNA 1 (DDX11-AS1) and AC092171.4 (Fig. 2). The characteristics of these 5 lncRNAs were listed in Table 1.

**Table 1:** Characteristics of the top 5 lncRNAs.

Gene symbol	Ensemble ID	Location	Log2 Fold change	AUC (95% CI)	p-value
HAGLR	ENSG00000224189	Chromosome 2: 176,173,195-176,188,958	5.650045829	0.9712 (0.9444-0.9980)	2.62E-05
SFTA1P	ENSG00000225383	Chromosome 10: 10,784,437-10,794,980	6.169656672	0.9646 (0.9228-1.0000)	2.64E-21
HAGLROS	ENSG00000226363	Chromosome 2: 176,177,717-176,179,008	5.002835084	0.9510 (0.9063-0.9957)	9.91E-13
DDX11-AS1	ENSG00000245614	Chromosome 12: 31,020,763-31,073,847	2.412997989	0.9596 (0.9249-0.9943)	0.000609851
AC092171.4	ENSG00000272953	Chromosome 7: 5,425,770-5,426,401	2.686680658	0.9564 (0.9212-0.9916)	5.20E-06

### Clinical value of these 5 aberrantly expressed lncRNAs in patients with HCC

**Figure 3:**
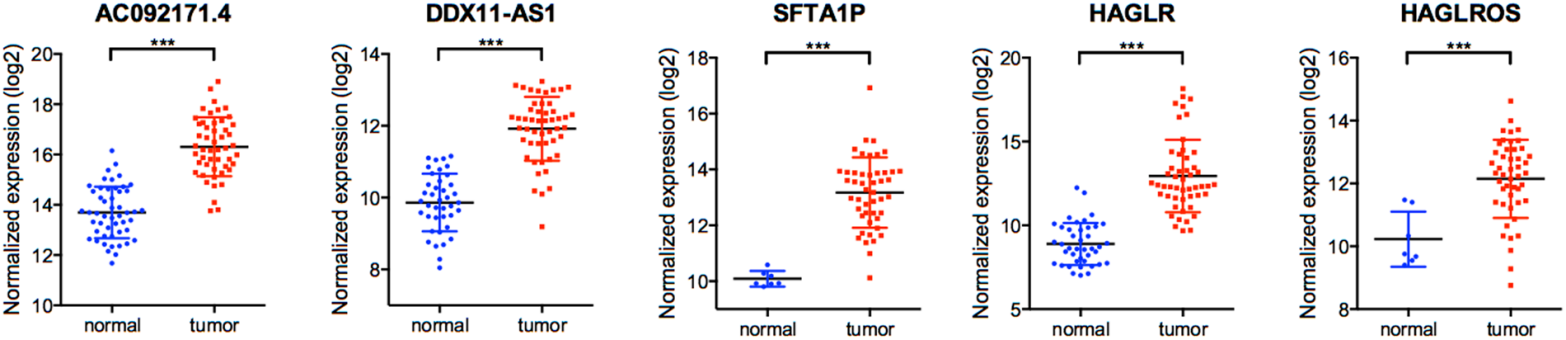
Expression of the top 5 lncRNAs between HCC and adjacent normal tissues. Data represent the mean ± SD, ^*^p < 0.05, ^**^p < 0.01, ^***^p < 0.001.

**Figure 4:**
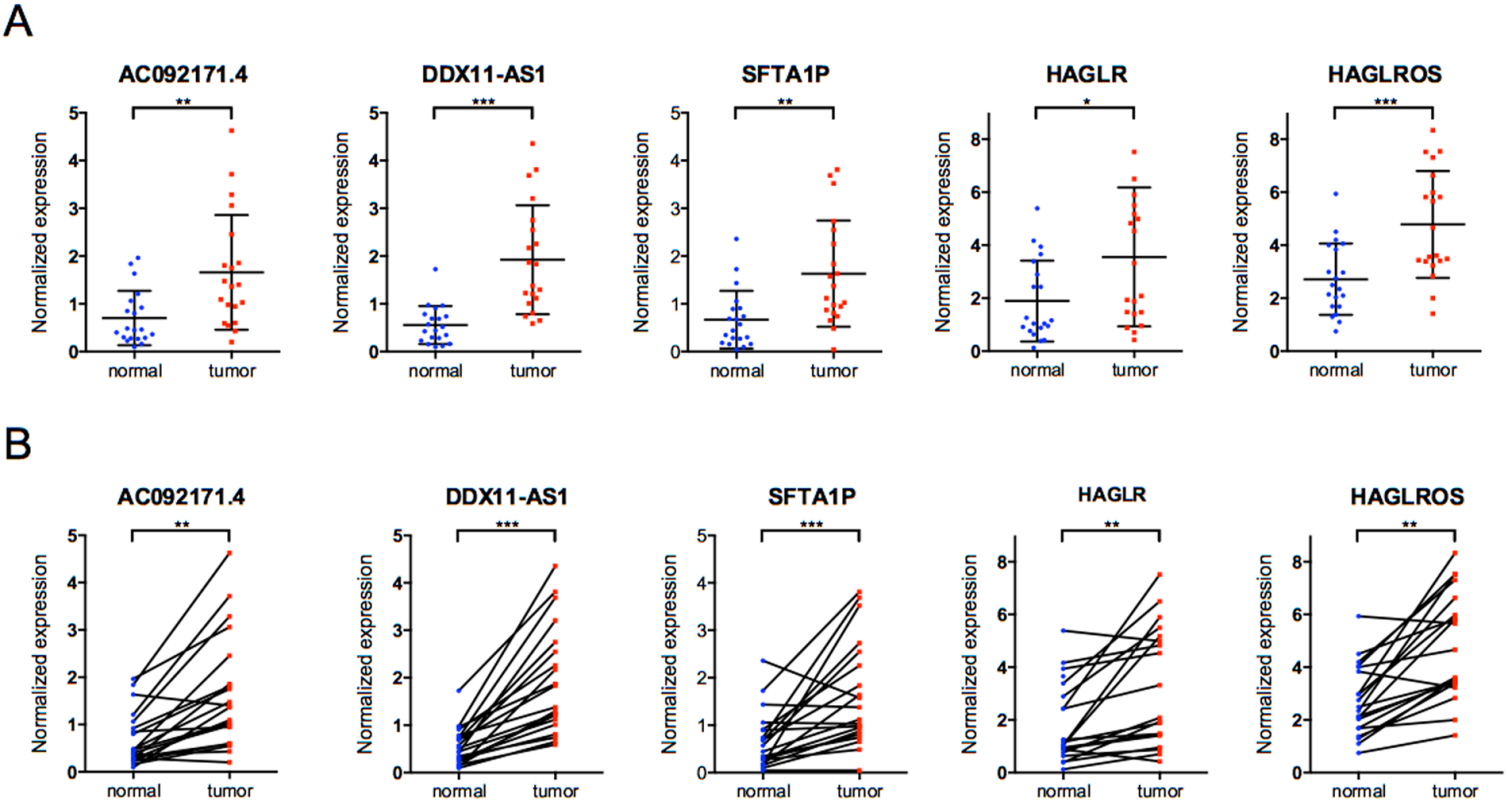
Validation of the expressions of the 5 lncRNAs with clinical samples. (**A**) The expression of the 5 lncRNAs in HCC and adjacent normal tissues, ^*^p < 0.05, ^**^p < 0.01, ^***^p < 0.001. (**A**) The expression of the 5 lncRNAs in HCC and paired-adjacent normal tissues, paired Student t test was used to assess the statistical significance, ^**^p < 0.01, ^***^p < 0.001.

**Figure 5:**
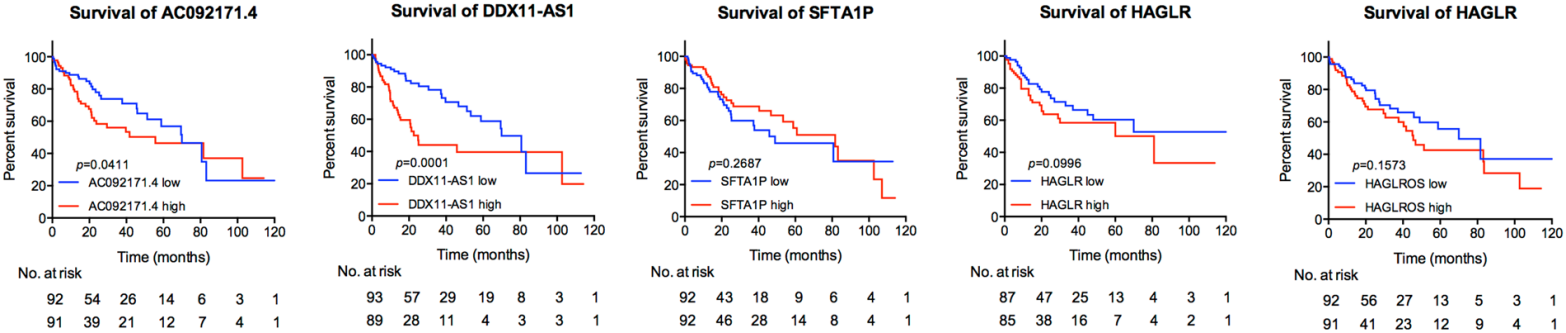
Kaplan-Meier analyses of OS based on the expressions of the top 5 lncRNAs.

Then the expression levels of the 5 lncRNAs between HCC and adjacent normal tissues were analyzed. Coincidentally, the expression levels of all the 5 lncRNAs were remarkably higher in HCC than normal tissues (Fig. 3). For the further validation on the distinguishing expression levels of these 5 lncRNAs between HCC and adjacent normal tissues, we performed qRT-PCR analysis using 20 HCC samples and paired normal tissues from West China Hospital. Comparing with normal tissues, notably higher expressions of all the 5 lncRNAs were observed in HCC (Fig. 4A,B). Kaplan-Meier curves showed that higher expressions of DDX11-AS1 and AC092171.4 were in correlation with poorer survival in HCC patients (*p* = 0.0001 and 0.0411, respectively), while the expressions of HAGLR, HAGLROS and SFTA1P were not associated with the HCC-specific survival (Fig. 5).

**Figure 6:**
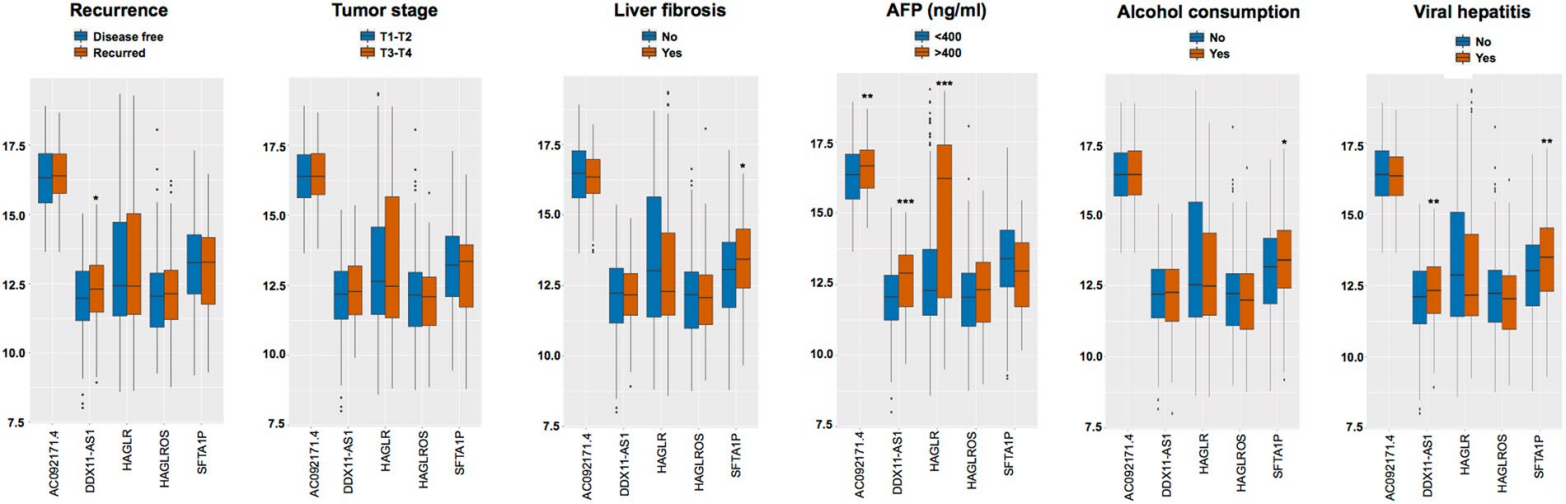
Association between the expression of the top 5 lncRNAs and clinicopathological parameters of patients with HCC. Data represent the mean (range), ^*^p < 0.05, ^**^p < 0.01, ^***^p < 0.001.

Moreover, correlation analysis between the expression of lncRNAs and clinicopathological parameters revealed that DDX11-AS1 was intimately associated with tumor recurrence (*p* < 0.05), tumor stage (*p* < 0.05), alpha fetoprotein (AFP) level (*p* < 0.0001) and viral hepatitis (*p* < 0.001). Meanwhile, higher expressions of HAGLR and AC092171.4 SFTA1P were observed in patients with high AFP level (>400 ng/ml). It was also found that patients with higher expression of SFTA1P tended to have liver fibrosis (*p* < 0.05), alcohol consumption (*p* < 0.05) and viral hepatitis (*p* < 0.01) (Fig. 6).

### Potential mechanism of the 5 aberrantly expressed lncRNAs in regulating the development and progression of HCC

**Figure 7:**
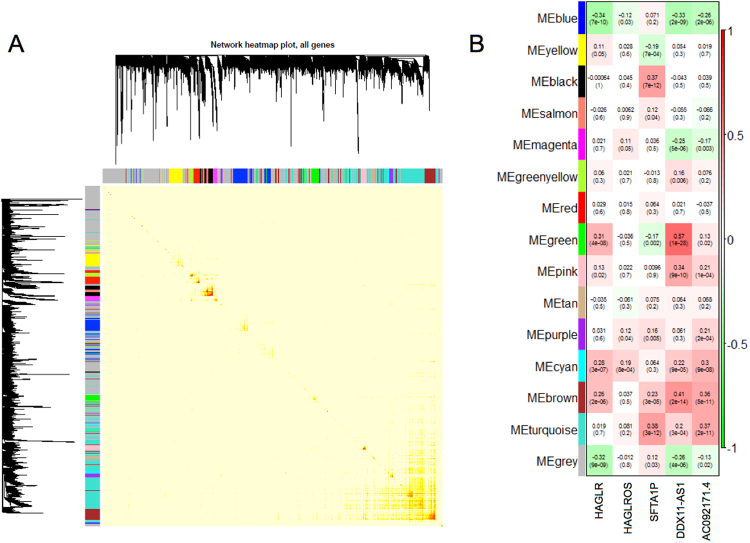
Gene co-expression network of the genes regulated by the 5 lncRNAs. (**A**) Pearson correlations between expression profiles of all pairs of genes were transformed into network connection strengths (denoted by intensity of red color). Average linkage hierarchical clustering with the topological overlap dissimilarity measure was used to identify gene co-expression modules, each of which was assigned a unique color. Rows and columns were symmetric and represent genes. (**B**) Correlation between module eigengenes and the expressions of the 5 lncRNAs. Each row corresponds to a module identified on the left side by its color. Each column corresponds to one lncRNA. Each cell reports the Pearson correlation between the module eigengene and lncRNAs using complete pairwise option along with uncorrected *p* value in parenthesis. Cells are color-coded using the correlation value according to color scale on the right; positive correlations are denoted red and negative correlation in green.

**Figure 8:**
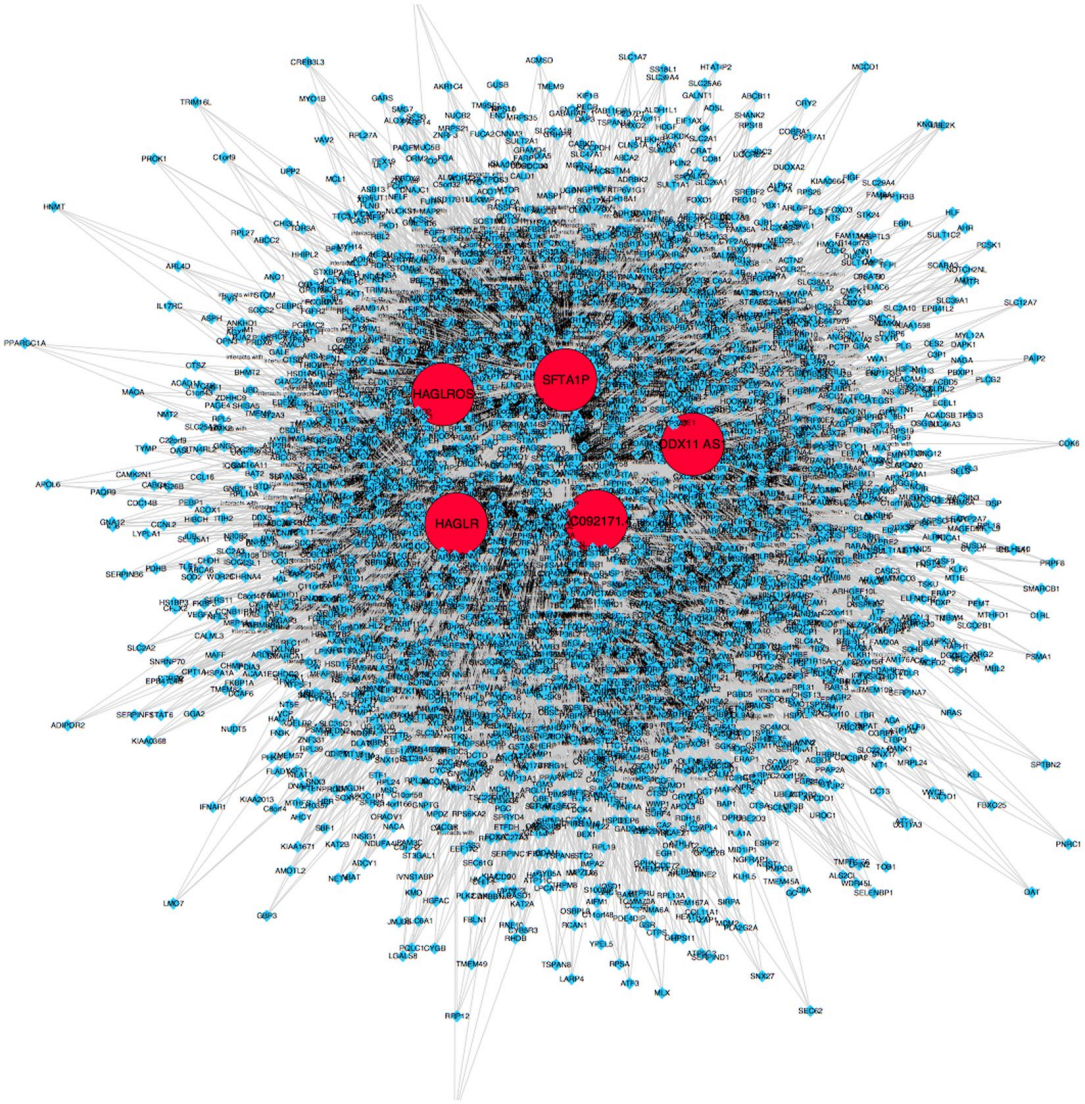
Visualization of the gene co-expression network of the 5 lncRNAs. Red balls represented the 5 lncRNAs, while the blue balls represented the co-expressed genes.

To explore the co-expressed genes and the potential functions of these 5 lncRNAs, we performed Weighted Gene Co-Expression Network Analysis (WCGNA) based on the expression profile of mRNAs in HCC from TCGA. Following quality control to remove technical outliers, 15 modules were identified by WGCNA. Each module was assigned a unique color identifier (Fig. 7A), with the remaining poorly connected genes colored gray. For instance, 6 modules are associated with HAGLR, including the blue, green, pink, cyan, brown and grey modules. However, only 3 modules, the blue, purple and cyan modules, are associated with HAGLROS (Fig. 7B). Meanwhile, the expression profile of mRNAs associated with the 5 lncRNAs was evaluated by Gene-Ontology (GO) analysis (Suppl Table 1). While the co-expressed genes of HAGLROS are mostly enriched in metabolic process (GO: 0008152), the co-expressed genes of HAGLR were significantly involved in cellular process (GO: 0009987). Regarding the potential pathways in regulating the progression of HCC, significant enrichment was observed in cholecystokinin receptors (CCKR) pathway and Gonadotropin-releasing hormone receptor (GNRHR) pathway, both of which belongs to G-protein coupled receptor (GPCR) related pathways. Moreover, genes correlated with the expressions of HAGLR and AC092171.4 were also enriched in the PI3K signaling pathway, which has been proved to play essential roles in the progression of cancer^14^. Visualization of the gene co-expression network of the 5 lncRNAs was shown in Fig. 8.

### Further analysis on the potential mechanism of DDX11-AS1 in regulating the development and progression of HCC

**Figure 9:**
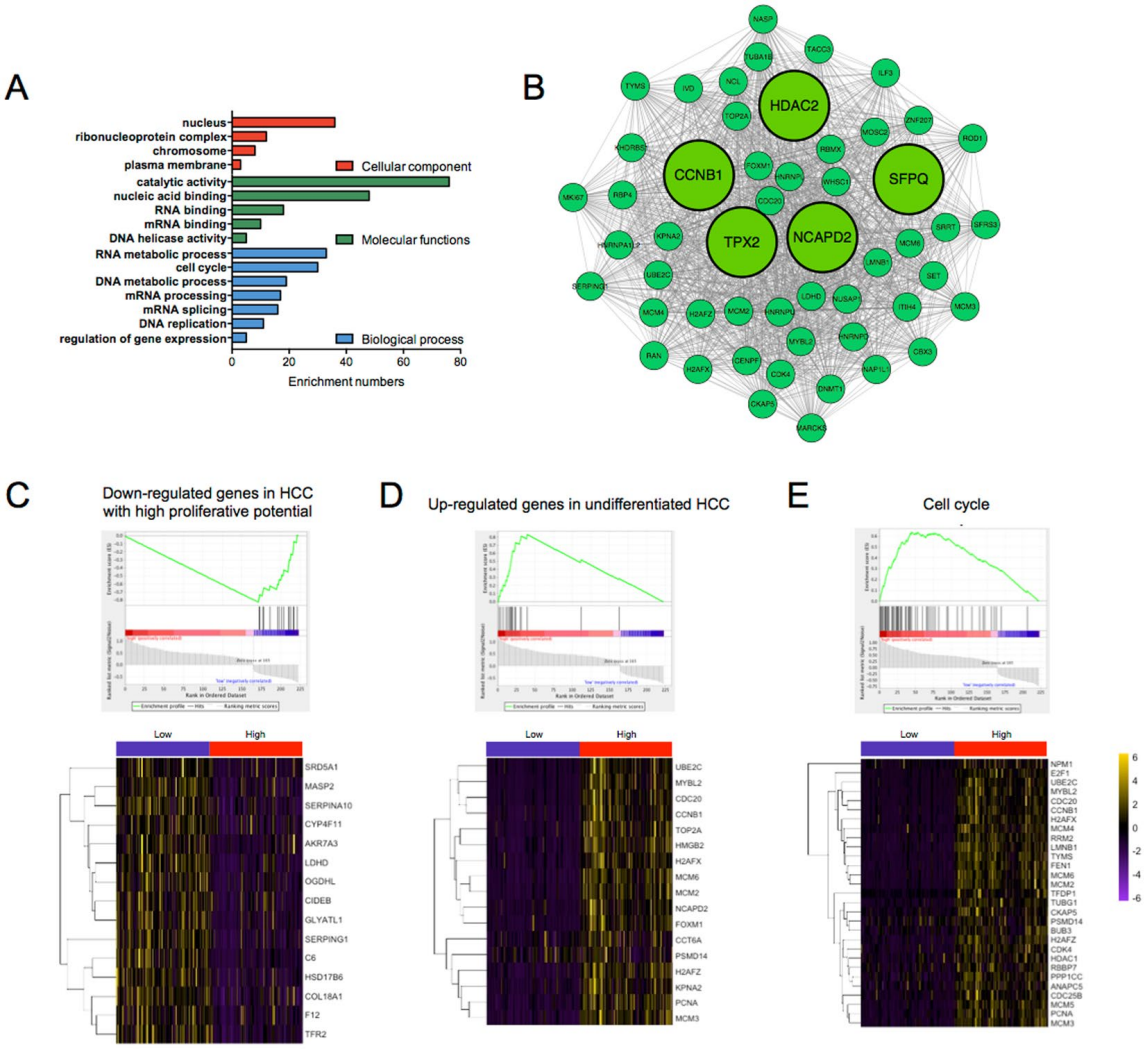
Association between DDX11-AS1 and the expressions of genes in green module. (**A**) GO analysis on genes within the green module. The x-axis indicates the numbers of enriched genes. (**B**) Visualization of hub genes within the green module. (**C**) Gene expression levels of the subsets of genes involved in HCC with high proliferative potency, HCC cell differentiation and cell cycle.

Among these 15 modules, significant correlation was observed between the green module and the expression of DDX11-AS1 (correlation > 0.50, *p* < 0.05, Fig. 6B). Therefore, further analysis was conducted on the mechanism of DDX11-AS1 in regulating the genes involved in green module. GO analysis of this module demonstrated that it was enriched in nucleus, catalytic activity and RNA metabolic process (Fig. 9A). Notable highly connected (“hub”) genes in this module include genes involved in transcriptional regulation (HDAC2 and SFPQ)^15,16^ and cell cycle (CCNB1, TPX2, NCAPD2)^17,18^, highly suggesting that this module might regulate the tumorigenesis of HCC (Fig. 9B). In order to determine the role of DDX11-AS1 in regulating the genes involved in this module, the Gene Set Enrichment Analysis (GSEA) was performed to analyze the gene expression profile affected by the expression level of DDX11-AS1 (high vs. low). The results showed that higher expression of DDX11-AS1 led to down-regulation of 15 genes (SRD5A1, CYP4F11, etc.), which have been proved to be down-regulated in HCC with high proliferative potential (*p* < 0.0001), suggesting that DDX11-AS1 might promote the proliferation of HCC by inhibiting the expressions of these 15 genes (Fig. 9C). Meanwhile, genes up-regulated in undifferentiated HCC were also enriched in patients with higher DDX11-AS1 expression (*p* < 0.001), indicating that DDX11-AS1 might participate in the process of HCC differentiation (Fig. 9D). Moreover, the expressions of genes involved in cell cycle were also increased in patients with higher DDX11-AS1 expression (*p* < 0.05, Fig. 9E). These findings suggested that DDX11-AS1 might promote the tumorigenesis of HCC by targeting the genes in the green module.

## Discussion

To date, numerous studies have been conducted to reveal the underlying mechanisms of lncRNAs in regulating the development and progression of HCC^19–21^. However, since the underlying mechanisms of HCC formation are complicated, studies focusing on a single genetic event may not meet the current requirement on finding potential molecular targets in treating HCC. In this study, we examined the lncRNA profiles of HCC tissues and paired adjacent normal tissues from TCGA dataset. 5 lncRNAs with the highest diagnostic accuracy (AUC >0.95) were identified and included for further analysis. The expressions of the 5 lncRNAs were verified in clinical HCC and paired normal tissues. The co-expression analysis showed that the 5 lncRNAs regulated the development and progression of HCC through various pathways, including p53 pathway and PI3 kinase pathway, leading to the occurrence of various biological processes. Moreover, more in-depth analysis on the mechanism of DDX11-AS1 demonstrated that DDX11-AS1 might regulate the proliferation and differentiation of HCC, indicating an essential role of DDX11-AS1 in hepatocarcinogenesis.

It has been widely known that the activation of PI3K pathway contribute to the progression of HCC^22^. The high-molecular-weight glycoprotein MUC15 can bind EGFR and accelerate EGFR internalization, then promotes EGFR degradation and inhibits EGF-induced PI3K-AKT activation, thus accelerating the process of HCC metastasis^23^. Through integrated bioinformatical analysis, we have identified two lncRNAs, HAGLR and AC092171.4, regulating the genes involved in PI3K signaling pathway, including AKT1, FOXO1, NRAS, PTEN, which has been previously reported to be the key regulators in the tumorigenesis and metastasis of HCC^23–26^. The activation of PI3K can phosphorylates and activates AKT, localizing it in the plasma membrane, leading to the localization of down-stream FOXO1 in the cytoplasm^27,28^. When dephosphorylated, FOXO1 can enter the nucleus and work as a transcription factor to promote cell cycle arrest at G1 phase via multiple mechanisms, including P27KIP1 and P130 up-regulation, and Cyclin D1 and D2 down-regulation^29^. It has been reported that ubiquitination and proteasome degradation of FOXO1 can lead to HCC progression, suggesting the role of FOXO1 as an onco-suppressor gene^24^. Similar with FOXO1, the PTEN gene is also identified as a tumor suppressor that is mutated in a large number of cancers at high frequency^30^. PTEN works by dephosphorylating PIP3 to PIP2, which limits AKTs ability to bind to the membrane, decreasing its activity^28^. The transcriptional activation of PTEN can suppress AKT-mTOR signaling, thus inhibiting the proliferation of HCC^31^. On the contrary, the NRAS is a well-known oncogene belonging to the Ras family, which consists of several distinct members including Ras (H, K, M, N, and R), Rap (1 and 2), and Ral. The Ras gene product is a membrane-bound guanosine triphosphate (GTP)/guanosine diphosphate (GDP)-binding (G) protein that serves as a “molecular switch”, converting signals from the cell membrane to the nucleus. These chemical signals lead to protein synthesis and regulation of cell survival, proliferation, and differentiation^32^. Liver cancer cells have mutations in the gene encoding the tumor suppressor protein p53 driven by NRAS become addicted to MYC stabilization, which enables the tumor cells to overcome a latent G2/M cell cycle arrest, thus promoting the proliferation of HCC^33^. These findings suggest the significance of genes involved in PI3K pathway in regulating the tumorigenesis and metastasis of HCC. Taken together, this study may inspire more research shedding light on the mechanism of how HAGLR and AC092171.4 modulate the expression of genes involved PI3K pathway in regulating the progression of HCC.

DDX11-AS1 is transcribed divergently from DDX11 promoter region, sharing with the protein-coding gene a common pattern of expression and regulation. Instead of affecting the levels of DDX11 mRNA or protein, DDX11-AS1 interacts with and regulates the activity of DDX11, a DNA dependent ATPase and helicase involved in DNA replication and sister chromatid cohesion, thus regulating DNA replication and sister chromatid cohesion^34^. In this study, patients with higher expressions of DDX11-AS1 tended to have poorer survival outcomes, suggesting that DDX11-AS1 can be a powerful predictor for survival of patients with HCC. Consistent with the previous work, the role of DDX11-AS1 in regulating DNA replication was also observed in current study, indicating that DDX11-AS1 is a key regulator in tumor cell proliferation. Moreover, the GSEA analysis showed that the expression of DDX11-AS1 affect the gene expressions involved in HCC proliferation, differentiation and cell cycle, which indicated an essential role of DDX11-AS1 as a promoter in the tumorigenesis of HCC, thus leading to the inferior survival outcomes. Therefore, understanding the novel RNA crosstalk between DDX11-AS1 and mRNAs, especially hub-genes in the green module, will probably lead to significant insight into gene regulatory networks, which have great implications in HCC development. Collectively, these data demonstrate the potential mechanistic links between an oncofetal lncRNA DDX11-AS1 and tumorigenesis, which established DDX11-AS1 as a potential therapeutic target in HCC.

In conclusion, we identified 1162 aberrantly expressed lncRNAs in HCC tissues and the top 5 of them were further analyzed to evaluate their clinical value as diagnostic and prognosis biomarkers. Meanwhile, analysis on the potential mechanisms of the 5 lncRNAs in regulating the development and progression of HCC showed a sophisticated network between lncRNAs and significant pathways, such as PI3K pathway. Moreover, we identified that DDX11-AS1 played vital roles in hepatocarcinogenesis, suggesting that DDX11-AS1 might serve as a prognostic biomarker and therapeutic target for HCC. These results require further validation studies on the mechanism of lncRNAs in manipulating HCC development.

## Electronic supplementary material

Supplementary Table 1

## References

[1] Chen W (2016). Cancer statistics in China, 2015. CA: a cancer journal for clinicians.

[2] Goh GB, Chang PE, Tan CK (2015). Changing epidemiology of hepatocellular carcinoma inAsia. Best practice & research. Clinical gastroenterology.

[3] Hoshida Y (2009). Integrative transcriptome analysis reveals common molecular subclasses of human hepatocellular carcinoma. Cancer research.

[4] Nakagawa S (2016). Molecular Liver Cancer Prevention in Cirrhosis by Organ Transcriptome Analysis and Lysophosphatidic Acid Pathway Inhibition. Cancer cell.

[5] Perkel JM (2013). Visiting “noncodarnia”. Bio Techniques.

[6] Grelet, S. *et al*. A regulated PNUTS mRNA to lncRNA splice switch mediates EMT and tumour progression. **19**, 1105–1115, 10.1038/ncb3595 (2017). 10.1038/ncb3595 PMC5578890 28825698

[7] Abraham JM, Meltzer SJ (2017). Long Noncoding RNAs in the Pathogenesis of Barrett’s Esophagus and Esophageal Carcinoma. Gastroenterology.

[8] Zhuo W, Kang Y (2017). Lnc-ing ROR1-HER3 and Hippo signalling in metastasis. Nature cell biology.

[9] Xu D (2013). Long noncoding RNAs associated with liver regeneration 1 accelerates hepatocyte proliferation during liver regeneration by activating Wnt/beta-catenin signaling. Hepatology (Baltimore, Md.).

[10] Huang JF (2013). Hepatitis B virus X protein (HBx)-related long noncoding RNA (lncRNA) down-regulated expression by HBx (Dreh) inhibits hepatocellular carcinoma metastasis by targeting the intermediate filament protein vimentin. Hepatology (Baltimore, Md.).

[11] Anders S, Huber W (2010). Differential expression analysis for sequence count data. Genome biology.

[12] Langfelder P, Horvath S (2008). WGCNA: an R package for weighted correlation network analysis. BMC bioinformatics.

[13] Subramanian A (2005). Gene set enrichment analysis: a knowledge-based approach for interpreting genome-wide expression profiles. Proceedings of the National Academy of Sciences of the United States of America.

[14] Fruman DA, Rommel C (2014). PI3K and cancer: lessons, challenges and opportunities. Nature reviews. Drug discovery.

[15] Schmidt DR, Schreiber SL (1999). Molecular association between ATR and two components of the nucleosome remodeling and deacetylating complex, HDAC2 and CHD4. Biochemistry.

[16] Patton JG, Porro EB, Galceran J, Tempst P, Nadal-Ginard B (1993). Cloning and characterization of PSF, a novel pre-mRNA splicing factor. Genes & development.

[17] Sartor H, Ehlert F, Grzeschik KH, Muller R, Adolph S (1992). Assignment of two human cell cycle genes, CDC25C and CCNB1, to 5q31 and 5q12, respectively. Genomics.

[18] Heidebrecht HJ (1997). p100: a novel proliferation-associated nuclear protein specifically restricted to cell cycle phases S, G2, and M. Blood.

[19] Yuan JH (2017). The MBNL3 splicing factor promotes hepatocellular carcinoma by increasing PXN expression through the alternative splicing of lncRNA-PXN-AS1. Nature cell biology..

[20] Wang Y (2015). The long noncoding RNA lncTCF7 promotes self-renewal of human liver cancer stem cells through activation of Wnt signaling. Cell stem cell..

[21] Yuan JH (2014). A long noncoding RNA activated by TGF-beta promotes the invasion-metastasis cascade in hepatocellular carcinoma. Cancer cell.

[22] Whittaker S, Marais R, Zhu AX (2010). The role of signaling pathways in the development and treatment of hepatocellular carcinoma. Oncogene.

[23] Wang RY (2013). MUC15 inhibits dimerization of EGFR and PI3K-AKT signaling and is associated with aggressive hepatocellular carcinomas in patients. Gastroenterology.

[24] Calvisi DF (2009). SKP2 and CKS1 promote degradation of cell cycle regulators and are associated with hepatocellular carcinoma prognosis. Gastroenterology.

[25] Matter MS (2016). Oncogenic driver genes and the inflammatory microenvironment dictate liver tumor phenotype. Hepatology (Baltimore, Md.).

[26] Meng F (2007). MicroRNA-21 regulates expression of the PTEN tumor suppressor gene in human hepatocellular cancer. Gastroenterology.

[27] King D, Yeomanson D, Bryant HE (2015). PI3King the lock: targeting the PI3K/Akt/mTOR pathway as a novel therapeutic strategy in neuroblastoma. Journal of pediatric hematology/oncology.

[28] Rafalski VA, Brunet A (2011). Energy metabolism in adult neural stem cell fate. Progress in neurobiology.

[29] Huang H, Tindall DJ (2007). Dynamic FoxO transcription factors. Journal of cell science.

[30] Song MS, Salmena L, Pandolfi PP (2012). The functions and regulation of the PTEN tumour suppressor. Nature reviews. Molecular cell biology.

[31] Li Y (2016). MAF1 suppresses AKT-mTOR signaling and liver cancer through activation of PTEN transcription. Hepatology (Baltimore, Md.).

[32] Adjei AA (2001). Blocking oncogenic Ras signaling for cancer therapy. Journal of the National Cancer Institute.

[33] Dauch, D. *et al*. A MYC-aurora kinase A protein complex represents an actionable drug target in p53-altered liver cancer. **22**, 744–753, 10.1038/nm.4107 (2016). 10.1038/nm.4107 27213815

[34] Marchese FP (2016). A Long Noncoding RNA Regulates Sister Chromatid Cohesion. Molecular cell.

