# Supplementary material for: Identification of The Aberrantly Expressed LncRNAs in Hepatocellular Carcinoma: A Bioinformatics Analysis Based on RNA-sequencing
Source: Sci Rep. 2018 Mar 29;8:5395. doi: 10.1038/s41598-018-23647-1 (PMC5876391; doi:10.1038/s41598-018-23647-1)
Supplement: Supplementary file 1 — Supplementary Table 1 [file 41598_2018_23647_MOESM1_ESM.docx]

**Identification of The Aberrantly Expressed LncRNAs in Hepatocellular Carcinoma: A Bioinformatics Analysis Based on RNA-sequencing**

**1, Hao-Tian Liao, M.D.**

Department of Liver Surgery, Liver Transplantation Division, West China Hospital, Sichuan University, Chengdu 610041, China

Email: liaohaotianmedmail@163.com

**2, Ji-Wei Huang, M.D.**

Department of Liver Surgery, Liver Transplantation Division, West China Hospital, Sichuan University, Chengdu 610041, China

Email: huangjiweimd@hotmail.com

**3, Tian Lan, M.D.**

Department of Liver Surgery, Liver Transplantation Division, West China Hospital, Sichuan University, Chengdu 610041, China

Email: 303344966@qq.com

**4, Jin-Ju Wang, M.D.**

Department of Liver Surgery, Liver Transplantation Division, West China Hospital, Sichuan University, Chengdu 610041, China

Email: [1448944361@qq.com](mailto:1448944361@qq.com)

**5, Bo Zhu, M.D.**

Department of Liver Surgery, Liver Transplantation Division, West China Hospital, Sichuan University, Chengdu 610041, China

Email: 1047406225@qq.com

**6, Corresponding Author：**

**Yong Zeng, Ph.D.**

Professor

Department of Liver Surgery, Liver Transplantation Division, West China Hospital, Sichuan University, Chengdu 610041, China

Email: zengyong@medmail.com.cn

**Ke-Fei Yuan, Ph.D.**

Associate Professor

Department of Liver Surgery, Liver Transplantation Division, West China Hospital, Sichuan University, Chengdu 610041, China

Email: ykf13@163.com

**Short title:** Bioinformatics analysis on the lncRNAs in HCC

**Type of manuscript:** original article

**Acknowledgments:** This work was supported by grants from the Natural Science Foundation of China (81770615, 81700555，81672882 and 81502441) and the Science and Technology Support Program of Sichuan Province (2017SZ0003). We thank the TCGA working group for generating publicly available data.

**Author Contributions:** Hao-Tian Liao and Ji-Wei Huang performed all the statistical analysis. Tian Lan and Jin-Ju Wang prepared Figures 1-9. Bo Zhu prepared Table 1 and Suppl Table 1. Hao-Tian Liao and Ji-Wei Huang co-wrote the manuscript. Yong Zeng and Kei-Fei Yuan conceived of the idea and designed the experiments. All authors have reviewed the manuscript.

**Conflicts of interest:** We declare no conflicts of interest concerning this article.

**Supplementary Table 1.** Significant GO terms based on the co-expressed genes of each lncRNA

| LncRNAs | Pathway | | | Biological process | | | Molecular function | | | Cellular component | | |
| --- | --- | --- | --- | --- | --- | --- | --- | --- | --- | --- | --- | --- |
|  | Term | n | *p* | Term | n | *p-* | Term | n | p-value | Term | n | *p-* |
| HAGLR | GNRHR pathway | 61 | 2.24E-03 | cellular process | 1252 | 1.66E-05 | catalytic activity | 1006 | 1.28E-41 | cell part | 903 | 8.56E-08 |
|  | CCKR signaling map | 54 | 3.46E-05 | metabolic process | 1192 | 6.48E-27 | hydrolase activity | 392 | 5.81E-10 | intracellular | 776 | 2.87E-12 |
|  | Apoptosis signaling pathway | 34 | 2.86E-02 | primary metabolic process | 1032 | 6.88E-23 | transferase activity | 249 | 1.36E-03 | cytoplasm | 507 | 5.26E-24 |
|  | p53 pathway | 28 | 3.23E-02 | protein metabolic process | 401 | 2.17E-12 | transporter activity | 205 | 7.13E-07 | organelle | 502 | 1.01E-06 |
|  | Blood coagulation | 25 | 1.15E-05 | localization | 374 | 1.54E-05 | oxidoreductase activity | 204 | 1.13E-30 | macromolecular complex | 296 | 1.50E-03 |
|  | PI3 kinase pathway | 21 | 9.09E-03 | transport | 340 | 6.38E-05 | transmembrane transporter activity | 168 | 3.58E-04 | cytosol | 139 | 2.96E-23 |
|  | Insulin/IGF pathway-protein kinase B signaling cascade | 17 | 1.59E-02 | biosynthetic process | 299 | 1.71E-04 | structural molecule activity | 166 | 4.98E-03 | ribonucleoprotein complex | 117 | 9.67E-13 |
|  | 5-Hydroxytryptamine degredation | 13 | 2.59E-03 | catabolic process | 184 | 9.08E-05 | DNA binding | 138 | 2.51E-04 | ribosome | 74 | 5.87E-20 |
|  | TCA cycle | 8 | 3.71E-02 | multicellular organismal process | 172 | 9.57E-09 | peptidase activity | 112 | 8.95E-06 | endoplasmic reticulum | 64 | 4.77E-06 |
|  |  |  |  | single-multicellular organism process | 171 | 1.65E-08 | sequence-specific DNA binding transcription factor activity | 100 | 1.15E-06 | mitochondrion | 48 | 1.94E-02 |
| HAGLROS | 5-Hydroxytryptamine degredation | 10 | 3.67E-08 | metabolic process | 220 | 5.47E-07 | catalytic activity | 211 | 2.76E-18 |  | | |
|  |  |  |  | primary metabolic process | 191 | 6.88E-06 | oxidoreductase activity | 73 | 1.40E-28 |  |  |  |
|  |  |  |  | lipid metabolic process | 55 | 1.35E-17 | transferase activity | 56 | 1.24E-02 |  |  |  |
|  |  |  |  | fatty acid metabolic process | 31 | 7.67E-16 | transporter activity | 45 | 4.53E-03 |  |  |  |
|  |  |  |  | cellular amino acid metabolic process | 31 | 1.91E-11 | transmembrane transporter activity | 41 | 3.04E-03 |  |  |  |
|  | Nicotine degradation | 4 | 2.04E-02 | carbohydrate metabolic process | 31 | 8.66E-05 | lyase activity | 17 | 4.66E-03 |  |  |  |
|  |  |  |  | ion transport | 22 | 1.08E-02 | receptor activity | 13 | 1.83E-02 |  |  |  |
|  |  |  |  | steroid metabolic process | 18 | 1.04E-05 | hydro-lyase activity | 8 | 1.01E-02 |  |  |  |
|  |  |  |  | cellular amino acid biosynthetic process | 15 | 1.44E-07 | racemase and epimerase activity | 7 | 4.02E-03 |  |  |  |
|  |  |  |  | coenzyme metabolic process | 14 | 1.42E-04 |  |  |  |  |  |  |
| SFTA1P | CCKR signaling map | 77 | 5.77E-09 | cellular process | 1688 | 2.32E-09 | catalytic activity | 1248 | 3.06E-35 | cell part | 1258 | 5.59E-17 |
|  | GNRHR pathway | 77 | 1.27E-03 | metabolic process | 1549 | 5.00E-29 | hydrolase activity | 512 | 1.06E-11 | intracellular | 1120 | 2.00E-30 |
|  | Integrin signalling pathway | 69 | 2.34E-04 | primary metabolic process | 1326 | 1.78E-22 | structural molecule activity | 252 | 6.71E-10 | organelle | 720 | 2.69E-16 |
|  | Huntington disease | 51 | 3.95E-03 | protein metabolic process | 535 | 8.01E-17 | transporter activity | 235 | 4.50E-03 | cytoplasm | 714 | 1.45E-41 |
|  | Parkinson disease | 38 | 1.32E-02 | localization | 506 | 1.12E-08 | oxidoreductase activity | 219 | 1.09E-20 | macromolecular complex | 444 | 5.60E-12 |
|  | Blood coagulation | 25 | 1.77E-03 | transport | 457 | 2.44E-07 | DNA binding | 180 | 1.06E-06 | integral to membrane | 169 | 2.42E-02 |
|  | Glycolysis | 15 | 1.83E-03 | response to stimulus | 452 | 1.15E-02 | receptor activity | 137 | 2.28E-12 | ribonucleoprotein complex | 165 | 2.45E-20 |
|  | Fructose galactose metabolism | 10 | 2.20E-02 | cellular component organization or biogenesis | 390 | 7.47E-03 | peptidase activity | 136 | 7.15E-05 | cytosol | 161 | 9.50E-21 |
|  |  |  |  | biosynthetic process | 388 | 7.14E-05 | sequence-specific DNA binding transcription factor activity | 136 | 1.77E-08 | ribosome | 95 | 1.79E-24 |
|  |  |  |  | catabolic process | 260 | 1.90E-09 | structural constituent of ribosome | 116 | 5.37E-23 | endoplasmic reticulum | 91 | 4.21E-10 |
| DDX11-AS1 | GNRHR pathway | 84 | 1.55E-04 | cellular process | 1760 | 8.25E-08 | catalytic activity | 1295 | 3.13E-44 | cell part | 1300 | 2.97E-14 |
|  | CCKR signaling map | 69 | 3.03E-05 | metabolic process | 1664 | 1.78E-35 | hydrolase activity | 490 | 3.62E-08 | intracellular | 1151 | 2.28E-26 |
|  | Integrin signalling pathway | 69 | 1.39E-03 | primary metabolic process | 1425 | 1.26E-27 | transferase activity | 331 | 8.96E-05 | cytoplasm | 747 | 5.49E-42 |
|  | Cytoskeletal regulation by Rho GTPase | 33 | 3.55E-02 | protein metabolic process | 556 | 4.39E-16 | oxidoreductase activity | 267 | 1.13E-38 | organelle | 733 | 5.22E-13 |
|  | Blood coagulation | 29 | 6.06E-05 | localization | 516 | 1.01E-06 | transporter activity | 254 | 9.22E-06 | macromolecular complex | 452 | 7.16E-10 |
|  | Ubiquitin proteasome pathway | 28 | 1.68E-02 | transport | 469 | 5.52E-06 | structural molecule activity | 233 | 4.21E-06 | plasma membrane | 218 | 2.02E-02 |
|  | De novo purine biosynthesis | 17 | 4.35E-02 | response to stimulus | 460 | 1.51E-04 | transmembrane transporter activity | 204 | 1.21E-02 | cytosol | 175 | 7.25E-24 |
|  |  |  |  | biosynthetic process | 420 | 1.65E-06 | DNA binding | 174 | 5.61E-08 | integral to membrane | 169 | 9.85E-04 |
|  |  |  |  | cellular component organization or biogenesis | 402 | 4.13E-02 | sequence-specific DNA binding transcription factor activity | 137 | 2.21E-08 | ribonucleoprotein complex | 166 | 2.04E-18 |
|  |  |  |  | catabolic process | 268 | 1.08E-08 | peptidase activity | 128 | 3.02E-03 | ribosome | 94 | 3.02E-22 |
| AC092171.4 | GNRHR pathway | 79 | 4.49E-04 | cellular process | 1661 | 1.66E-06 | catalytic activity | 1295 | 3.13E-44 | cell part | 1250 | 1.88E-15 |
|  | CCKR signaling map | 69 | 4.63E-06 | metabolic process | 1577 | 4.33E-33 | hydrolase activity | 490 | 3.62E-08 | intracellular | 1109 | 7.71E-28 |
|  | Integrin signalling pathway | 65 | 3.14E-03 | primary metabolic process | 1344 | 1.05E-24 | transferase activity | 331 | 8.96E-05 | cytoplasm | 732 | 6.02E-46 |
|  | Ubiquitin proteasome pathway | 26 | 4.05E-02 | protein metabolic process | 529 | 1.99E-15 | oxidoreductase activity | 267 | 1.13E-38 | organelle | 705 | 1.19E-13 |
|  | Blood coagulation | 25 | 1.87E-03 | localization | 488 | 4.29E-06 | transporter activity | 254 | 9.22E-06 | macromolecular complex | 440 | 4.27E-11 |
|  | PI3 kinase pathway | 25 | 1.21E-02 | nitrogen compound metabolic process | 475 | 2.75E-02 | structural molecule activity | 233 | 4.21E-06 | cytosol | 173 | 1.81E-25 |
|  | 5-Hydroxytryptamine degradation | 13 | 4.52E-02 | transport | 446 | 1.01E-05 | transmembrane transporter activity | 204 | 1.21E-02 | integral to membrane | 163 | 3.51E-03 |
|  | Cholesterol biosynthesis | 10 | 4.24E-02 | response to stimulus | 446 | 2.33E-03 | DNA binding | 174 | 5.61E-08 | ribonucleoprotein complex | 161 | 1.05E-18 |
|  |  |  |  | biosynthetic process | 412 | 4.95E-08 | sequence-specific DNA binding transcription factor activity | 137 | 2.21E-08 | ribosome | 98 | 3.90E-26 |
|  |  |  |  | catabolic process | 259 | 4.17E-09 | peptidase activity | 128 | 3.02E-03 | endoplasmic reticulum | 91 | 4.97E-10 |
